# Fenugreek Seed Galactomannan Aqueous and Extract Protects against Diabetic Nephropathy and Liver Damage by Targeting NF-κB and Keap1/Nrf2 Axis

**DOI:** 10.3390/toxics10070362

**Published:** 2022-06-30

**Authors:** Sarah M. Alsuliam, Nawal A. Albadr, Salah A. Almaiman, Abdullrahman S. Al-Khalifah, Noorah S. Alkhaldy, Ghedeir M. Alshammari

**Affiliations:** 1 Department of Food Science and Nutrition, College of Food and Agricultural Sciences, King Saud University, Riyadh 11451, Saudi Arabia; 438203832@student.ksu.edu.sa (S.M.A.); smaiman@ksu.edu.sa (S.A.A.); akhalifa@ksu.edu.sa (A.S.A.-K.); aghedeir@ksu.edu.sa (G.M.A.); 2 Department of Community Health Sciences, College of Applied Medical Sciences, King Saud University, Riyadh 11451, Saudi Arabia; 441203164@student.ksu.edu.sa

**Keywords:** Fenugreek seeds, T1DM, oxidative stress, inflammation, liver, kidneys, lipids

## Abstract

This investigation was conducted to test the potential of the galactomannan (F-GAL) and aqueous extract (FS-AE) of the Fenugreek seed aqueous to prevent liver and kidney damage extracts in streptozotocin (STZ)-induced T1DM in rats. Non-diabetic and diabetic rats received the normal saline as a vehicle or were treated with FS-EA or F-GAL at a final concentration of 500 mg/kg/each. Treatments with both drugs reduced fasting hyperglycemia and improved serum and hepatic lipid profiles in the control and diabetic rats. Additionally, F-GAL and FS-AE attenuated the associated reduction in the mass and structure of the islets of Langerhans in diabetic rats and improved the structure of the kidneys and livers. In association, they also reduced the generation of reactive oxygen species (ROS), lipid peroxides, factor (TNF-α), interleukin-6 (IL-6), and nuclear levels of NF-κB p65, and improved serum levels of ALT, AST, albumin, and creatinine. However, both treatments increased hepatic and renal superoxide dismutase (SOD) in the livers and kidneys of both the control and diabetic-treated rats, which coincided with a significant increase in transcription, translation, and nuclear localization of Nrf2. In conclusion, FS-AE and F-GAL are effective therapeutic options that may afford a possible treatment for T1DM by attenuating pancreatic damage, hyperglycemia, hyperlipidemia, and hepatic and renal damage.

## 1. Introduction

Diabetes mellitus (DM) is a prodigious chronic disorder that results from absolute/relative loss of insulin (type-1 DM) or impaired insulin action and signal transduction (T2DM) [1]. Additionally, hyperglycemia associated with T1DM and T2DM is a leading cause of macro-and microvascular complications, as well as multi-organ damage, including nephropathy, neuropathy, retinopathy, and liver damage [2,3]. Regarding the liver, T1DM and T2DM are associated with impaired hepatic lipid metabolisms, which induce hyperlipidemia and hepatic steatosis, eventually leading to the development of non-alcoholic fatty liver disease [4,5]. In addition, T1DM and T2DM are leading causes of liver and kidney damage by provoking oxidative stress, inflammation, fibrosis, and apoptosis [6,7,8].

Nonetheless, the overproduction of reactive oxygen species (ROS) was a major mechanism leading to hepatic and renal damage in diabetic subjects and animals by provoking all the other damaging pathways, including inflammation, fibrosis, and apoptosis [2]. Indeed, hyperglycemia alone can trigger massive quantities of ROS in the livers and kidneys through several mechanisms, including glucose auto-oxidation, mitochondria damage, and activation of several pathways, including the protein kinase C (PKC), advanced-glycation end-products (AGEs), polyol, and hexosamine phosphate pathway [9]. On the other hand, ROS promotes liver and kidney damage by depleting the antioxidant stores, activating the nuclear factor kappa-B (NF-κB), a master inflammatory transcription factor that upregulates inflammatory cytokines production, and upregulating the transforming growth factor-β1 (TGF-β1) signaling which stimulates collagen deposition [6,10,11]. In addition, ROS can induce DNA damage and activate intrinsic cell apoptosis by upregulating the 53/Bax axis [12,13].

In the majority of cells, including the liver and renal cells, the nuclear factor erythroid 2 related factor-2 (Nrf2) is the major transcription responsible for boosting cell antioxidant potential by stimulating the expression of synthesis of glutathione (GSH) and phase-II antioxidant enzymes such as heme oxygenase-1 (HO-1), catalase (CAT), glutathione peroxidase (GPX), and superoxide dismutase (SOD) [11,14]. In addition, Nrf2 is a potent inflammatory factor that can suppress inflammation by inhibiting the activation of NF-κB and the NRLP3 inflammasome [11,15]. Furthermore, Nrf2 can inhibit cell apoptosis by upregulating anti-apoptotic proteins such as Bcl2 [15]. Interestingly, the transcriptional activity of Nrf2 is tightly regulated by its cytoplasmic interaction with the Kelch-like ECH-associated protein 1 (keap-1), which normally stimulates its proteasome-mediated degradation in the cytoplasm [11]. However, ROS can phosphorylate keap-1, thus releasing Nrf2, which is then translocated to the nucleus to initiate the transcription process [11,16,17]. Of note, accumulating data have shown that DM induces damage in a variety of organs by depleting the levels and the transcriptional activation of Nrf2, as well as reducing the expression of antioxidants [7,8,9,10,11,12,13]. Opposing this, pharmacological or transgenic activation of Nrf2 in experimental DM attenuated retinopathy, nephropathy, neuropathy, atherosclerosis, and liver damage by suppressing oxidative stress, inflammation, and apoptosis and independent of modulating hyperglycemia [10,11,17,18,19]. These data suggest that any drug that is able to stimulate the Nrf2/antioxidant axis may present a novel therapy to alleviate DM-induced complications.

On the other hand, the research on anti-hyperglycemic and anti-diabetic drugs from the plant kingdom is extensively increasing with well-reported findings [20,21]. Fenugreek (*Trigonella foenum-graecum* L.) is a plant member of the Fabaceae family that grows globally and is traditionally used as spices and dietary supplements [22]. Different extracts from Fenugreek have shown promising protective effects against liver and renal damage in animal models and human subjects due to its potent antioxidant, anti-inflammatory, and anti-apoptotic powers, mainly mediated by scavenging ROS and upregulation of antioxidants [23,24]. Other reported biological activities of Fenugreek seed (FS) extract included being an effective therapy to alleviate ulcers, immune disorders, and cancer [23]. The various extract obtained from FS also has potent anti-diabetic, hypoglycemic, insulinotropic, and hypolipidemic potentials in both human and animal models of T1DM and T2DM [22,25,26,27]. Placebo subjects are three to four times more at risk of developing T2DM compared with subjects who received Fenugreek seed extract [27]. Interestingly, the hypoglycemia afforded by FS was reported to include various mechanisms, including suppressing glucose absorption, inhibiting hepatic gluconeogenesis, stimulating peripheral insulin sensitivity, and stimulating the generation of pancreatic β-cells [28,29]. With respect to its protective effects against DM-mediated organ damage, FS aqueous extract (FS-AE) prevented STZ-induced diabetic nephropathy by reducing circulatory levels of HbA1c, decreasing ROS generation, attenuating the increase in transforming growth factor-β1 (TGF-β1) and collagen deposition, and stimulating sulfated glycosaminoglycans [30,31]. Treatment with glycosides-based standardized Fenugreek seed extract also suppressed pulmonary inflammation, fibrosis, and apoptosis by activating Nrf2-mediated suppression of NF-κB and Bax and repressed cisplatin-induced testicular damage and apoptosis by inhibiting NF-κB [32,33].

Galactomannan (GAL) is the primary soluble dietary fiber isolated from Fenugreek seed endosperm. Several lines of evidence have also shown that Fenugreek GAL (F-GAL) is a potential therapy to manage T1DM and T2DM and their complications [34,35,36]. Like FS extract, the mechanisms by which F-GAL exerts its hypoglycemic effect include suppressing intestinal glucose absorption, inhibiting hepatic glucose production, promoting the generation of pancreatic β-cells, improving glucose resistance, suppressing oxidative stress, lipid peroxidation, and inflammation [35]. Additionally, F-GAL inhibited lipid peroxidation and stimulated antioxidant enzymes in the livers of diabetic rats [37].

Despite these reported hypoglycemic effects of both FS and F-GAL, their protective potential against DM-mediated liver and kidney injury is still not established. Therefore, this study was conducted with two major objectives. First, to compare the renal and hepatic protective effects of FS-AE and F-GAL in streptozotocin (STZ)-induced T2DM in rats. Second, to examine some protective mechanisms by targeting their effects on glucose/insulin levels, structures of pancreatic β-cells, levels of antioxidants, and expression/activity of Nrf2 and NF-κB.

## 2. Materials and Methods

### 2.1. Animals

Adult male Wistar rats (220 ± 15 g) were provided from the Experimental Animal Care Center at King Saud University, Riyadh, KSA, and were maintained there during the treatment period. During this period, all animals were housed in a temperature and humidity-controlled room (21 ± 1 °C and 55–65%). The light/dark was automatically switched every 12 h. All rats had free access to diets and drinking water. All procedures were conducted after obtaining ethical approval from the Research Ethics Committee (REC) at King Saudi University, Riyadh, Saudi Arabia (Ethical Reference No: KSU-SE-22-08).

### 2.2. Induction of Diabetes Mellitus

T1DM Induction was introduced to desired animals as previously described in our studies [38] using an intraperitoneal dose of STZ (65 mg/kg) freshly prepared in 0.5 M sodium citrate buffer (pH 4.5). The animals were administered 0.5 glucose during the first 2 days to prevent sudden hypoglycemia and then monitored for 10 days. After this period, glucose was measured, and all animals with blood glucose higher than 300 mg/dL were identified as diabetic rats and included in the experimental procedure.

### 2.3. Preparation of the Fenugreek Seeds Aqueous Extract (FS-AE)

Fenugreek seeds were purchased from a certified supplier from Riyadh, KSA, and were identified and taxonomically authenticated at the Department of Pharmacognosy, College of Pharmacy, King Saud University, Riyadh, KSA. To prepare for the FS-AE, we have followed the protocols published by others [39]. Briefly, 500 g were soaked in 5 L distilled water and boiled for 30 min. The extract was then filtered, dried under vacuum, and then lyophilized. This protocol yields 3.6%, which equals 18 g.

### 2.4. Preparation and Isolation of F-GAL

This followed the protocol described by Jiang et al. [35]. In brief, 500 g of Fenugreek were grounded and extracted 1 h at 60 °C in 20 volume petroleum ether to remove all lipids. The resulting residue (430 g) was then vacuum dried and soaked for 90 min in 10 volumes of 70% ethanol to get rid of all alcohol-soluble proteins, glycosides, oligosaccharides, and monosaccharides. Ethanol was then dried under vacuum, and the new resultant residue 310 g was extracted 4 times in 50 volume water for 60 min at 10 °C and then centrifuged for 11,000× *g* for another 20 min. The resultant supernatant was collected and mixed with absolute ethanol (3:7, *v*/*v*). The whole mixture was then incubated overnight at 4 °C, and F-GAL was precipitated. The precipitate was removed, dissolved in water, and then lyophilized. This protocol yielded about 30% of F-GAL (140 g).

### 2.5. Experimental Design

Control and diabetic animals were randomly selected and distributed into six groups (each of eight rats) as follows: (1) control: normal rats administered an equal volume of normal saline as a vehicle; (2) control + FS-AE: normal rats administered FS-AE at a final dose of 500 mg/kg; (3) control + F-GAL: control rats administered F-GAL at a final dose of 500 mg/kg; (4) T1DM: rats with pre-established STZ-T1DM and administered an equivalent volume of normal saline as a vehicle; (5) T1DM + FS-AE: rats with pre-established T1DM and administered FS-AE at a final dose of 500 mg/kg; (6) T1DM + F-GAL: rats with pre-established T1DM and administered F-GAL at a final dose of 500 mg/kg. All treatments were given orally using gavage for 8 weeks, which was shown by others to induce hepato-renal damage in rats using the selected dose of STZ [40].

### 2.6. Dose Selection

The selected dose of FS-AE was based on the study of Berroukche et al. [39], which has shown the hypoglycemic and hypolipidemic effect of this extract in rats, whereas the selected dose of F-GAL was based on the study of Jiang et al. [35], who demonstrated a potent hypoglycemic effect of this dose in rats and suggested that it has antioxidant and anti-inflammatory effects.

### 2.7. Urine and Blood Tissue Collection and Processing

By the end of the last treatment in week 8, the animals were fasted overnight and then anesthetized by an i.p. bolus of ketamine/Xylazine hydrochloride (90:10 mg/kg). Blood samples (1 mL) were directly collected from the heart into either EDTA or plain tubes, centrifuged (3000 rpm/10 min), and supernatants (plasma and serum) were separated and preserved at −20 °C as serum. The animals were then ethically authenticated by cervical dislocation. Their abdomens were opened. Their kidneys, livers, and pancreases were collected on ice and washed with ice-cold phosphate buffer saline (PBS) (pH = 7.4). These tissues were cut into small pieces, some fixed in 10% buffered formalin or snap-frozen in liquid nitrogen and preserved at −80 °C.

### 2.8. Measurement of Glucose and Insulin Levels

Plasma glucose and insulin levels were measured using assay and ELISA kits, respectively (#10009582; Cayman Chemical, Ann Arbor, MI, USA and #589501, Ann Arbor, MI, USA). The HOMA-beta cell function (HOMA-β) was calculated by using the following formula: 360 × fasting insulin (μU/mL)/(fasting glucose (mg/dL) − 63) [41].

### 2.9. Analysis of Kidney and Liver Functions

Serum levels of urea were measured using colorimetric assay kits (#DIUR-100 BioAssay Systems, Hayward, CA, USA). Serum and urine creatinine (Cr) and albumin levels were measured using a commercially available kit (#MBS841754, MyBioSorces, San Diego, CA, USA). Serum levels of liver function enzymes, including Alanine aminotransferase (ALT) and aspartate aminotransferase (AST), were measured using rats’ specific ELISA assay kits (cat MBS269614 and MBS264975; respectively, MyBioSource, San Diego, CA, USA). All protocols were conducted per each kit’s instructions for *n* = 8 rats/group.

### 2.10. Preparation of Liver Homogenates and Biochemical Analysis

Samples (100 mg) of the frozen liver and kidney tissues were homogenized in 0.5 mL ice-cold PBS (pH = 7.4), which was then centrifuged at 1200× *g* to isolate the supernatant containing the tissue homogenates. The supernatants were frozen at −80 °C and used later to measure levels of the following oxidative stress, and inflammation-related markers using rat-specific assay or ELISA kits. The levels of ROS/RNS in tissue lysate were measured using a DCF-based kit (#ab238535; Abcam, Cambridge, CB2 0AX, UK) and as previously used by others [42,43,44]. Homogenates levels of malondialdehyde (MDA) were measured using special rat assay kits (#10009055, Cayman, Ann Arbor, MI, USA) as validated previously in our labs and described previously by others [45,46,47,48]. The other rat-specific ELISA kits were used to measure the homogenate levels of superoxide dismutase (SOD) (#MBS036924, MyBioSource, San Diego, CA, USA); tumor necrosis factor-alpha (TNF-α) (#BMS622, ThermoFisher, Waltham, MA, USA), and interleukine-6 (IL-6) (#R6000B, R&D System, Minneapolis, MN, USA). Total cytoplasmic levels of keap-1 in the total cell homogenates were measured using a rat-specific ELISA kit (#MBS7218529, MyBioSource, San Diego, CA, USA). The nuclear extracts from the frozen liver and kidney tissues were prepared using a special isolation kit and as per the manufacturer’s instruction (ab113474, Abcam, Cambridge, CB2 0AX, UK). The nuclear and cytoplasmic levels of NF-κB p65 and Nrf2 were measured using ELISA kits (Cat. No. MBS2505513; Cat. No. MBS752046; MyBioSource, San Diego, CA, USA). All protocols were conducted per each kit’s instructions for *n* = 8 samples/group.

### 2.11. Extraction of Hepatic Lipids

Hepatic lipids were extracted from the frozen livers (*n* = 6 liver samples/group) as described by the method of Folch et al. [49]. Part of the liver tissue (0.5 g) was homogenized in 20-volume (20 mL) of 1:2 *v*/*v* methanol: chloroform mixture for 4–5 h. Then, 4 mL of normal saline were added to each tube and vortexed at 2500× *g* for 15 min to separate the layers. The lower layer containing the lipid fraction was separated, and the solvent was evaporated. The accumulated lipids were dissolved in 1 mL isopropanol and used later to determine the lipid profile. All parameters were analyzed for *n* = 6 samples/group and per kit instruction.

### 2.12. Lipid Profile in the Serum and Livers

Hepatic and serum levels of cholesterol were determined using the multi-enzyme-based kit (#ECCH-100, BioAssay Systems, Hayward, CA, USA). Serum and hepatic triglycerides were determined using a colorimetric assay kit (#10010303, Cayman Chemical, Ann Arbor, MI, USA). Serum and hepatic levels of high-density lipoprotein cholesterol (HDL-c) were determined using an assay kit (#K4436, BioVision, Milpitas, CA, USA). Serum and hepatic levels of low-density lipoprotein cholesterol (LDL-c) were determined using an assay kit (#79960, Crystal Chemicals, Elk Grove Village, IL, USA). All parameters were analyzed for *n* = 6 samples/group and per each kit instruction.

### 2.13. Quantitate Real-Time Polymerase Chain Reaction (q-PCR)

qPCR was conducted to measure the total cytoplasmic mRNA level of some target genes, including Nrf2, keap-1, and β-actin in rats. All primers were purchased from ThermoFisher Scientific, Waltham, MA, USA, and are described in Table 1. Total RNA and cDNA were prepared to form the frozen renal and hepatic tissues using commercial kits (#74004, Qiagen, Germany and #K1621, ThromoFisher Scientific, Waltham, MA, USA, respectively). The amplification was conducted using a BioRad CFX96 thermal cycler (USA). The 20 µL reaction mixture contained the following ingredients: 2 μL cDNA (final concentration = 500 ng/µL), 0.4 μL of both primers forward and reverse primers (final concentration = 200 nm/each), 10 μL of the Ssofast Evergreen Supermix (#172-5200, BioRad, Hercules, CA, USA), and 7.2 μL RNase-free water. The amplification steps were (1) a heating cycle (95 °C/30 s); (2) 40 cycles of denaturation (95 °C/5 s), (3) 40 cycles of primers annealing and extension (60 °C/60 s); and (4) a final melting step at 95 °C/30 s). Normalization was performed according to the ∆∆CT method using the provided software of the PCR and against the loading control. All procedures were conducted according to the manufacturer’s instructions and *n* = 8 samples/groups.

### 2.14. Histological Evaluation

The 24 formalin-fixed liver tissues were then dehydrated in ascending concentration of alcohol (70–100%), cleared with xylene, embedded in paraffin, and cut into a 3 μm section. The tissues were routinely stained with hematoxylin and eosin (H&E) and examined under a light microscope (Model Nikon eclipse E200, Tokyo, Japan) by a blind pathologist unaware of any experimental groups.

### 2.15. Statistical Analysis

GraphPad Prism statistical software (Sydney, Australia) was used for the analysis of the data. The homogeneity of variance and normality of the data were tested using the Levene’s test and Kolmogorov–Smirnov test, respectively. The significance of the data was tested using the 1-way ANOVA analysis, where Tukey’s *t*-test was used as post hoc. All values were presented as means + standard deviations (SD) and were considered significantly varied to *p* < 0.05.

## 3. Results

### 3.1. Changes in Metabolic Parameters

Final liver, kidney, and body weights, last 4 weeks’ food intake, and fasting insulin levels were not significantly different but fasting blood glucose was significantly lower, and the levels of HOMA-β were significantly higher in both FS-AE and F-GAL-treated rats as compared to control rats (Table 2). The reduction in fasting glucose levels and the increment in the value HOMA-β were significant in F-GAL-treated rats as compared to FS-AE-treated rats (Table 2). However, final body and liver weights, average last 4 weeks’ food intake, and fasting glucose levels were significantly increased, whereas kidney weights, fasting insulin levels, and values of HOMA-β were significantly increased in STZ-diabetic rats as compared to control, FS-AE, and F-GAL-treated rats (Table 2). The levels of all these parameters were significantly reversed in STZ + FS-AE and STZ + F-GAL-treated rats as compared to STZ-diabetic model rats (Table 2). Additionally, the improvements in the levels of all these markers were more profound in STZ + F-GAL when compared to STZ + FS-AE-treated rats (Table 2). Although the levels of all these measures were not significantly varied, levels of fasting glucose, fasting insulin, and HOMA-β did not reach their basal levels when a comparison was made between STZ + F-GAL and control rats (Table 2).

### 3.2. Changes in Serum and Hepatic Lipids

Levels of all measured serum and hepatic lipids did not vary significantly between the control, FS-AE, and F-GAL-treated rats (Table 3). Serum levels of HDL-c were significantly decreased, but serum and hepatic levels of TGs, CHOL, and FFAs, as well as serum levels of LDL-c, were significantly increased in STZ-model rats as compared to control rats (Table 3). On the opposite, serum levels of HDL-c were significantly increased, but serum and hepatic levels of TGs, CHOL, and FFAs, as well as serum levels of LDL-c, were significantly decreased in both STZ + FS-AE and STZ + F-GAL as compared to STZ-diabetic rats (Table 3). Additionally, levels of HDL-c were significantly higher, but the levels of all other biochemical endpoints were significantly lower in STZ + GAL-treated rats as compared to STZ + FS-AE. Yet, although the levels of the majority of the lipids were not significantly different between the STZ + F-GAL and control rats, the serum and hepatic levels of TGs and FFAs remained significantly higher in the STZ + F-GAL-treated rats (Table 3).

### 3.3. Changes in Serum Hepatic and Renal Function Markers

No significant differences in the serum levels of ALT, AST, urea, creatinine, and albumin were depicted when a comparison was made between the control, FS-AE, and F-GAL-treated rats (Table 4). The levels of ALT, AST, urea, and creatinine were significantly increased, but the levels of serum albumin were significantly decreased in STZ-treated diabetic rats as compared to control rats (Table 4). These events were reversed in both the STZ + FS-AE and STZ + F-GAL-treated rats as compared to STZ-treated rats (Table 4). In addition, the reduction in ALT, AST, urea, and creatinine levels and the increase in the levels of albumin were significantly more in the serum of STZ + F-GAL-treated rats than in control rats. Despite this, the levels of ALT, AST, and creatinine did not reach their basal levels in STZ + F-GAL as compared to control rats (Table 4).

### 3.4. Changes in Hepatic and Renal Oxidative Stress/Antioxidant Markers

**Figure 1:**
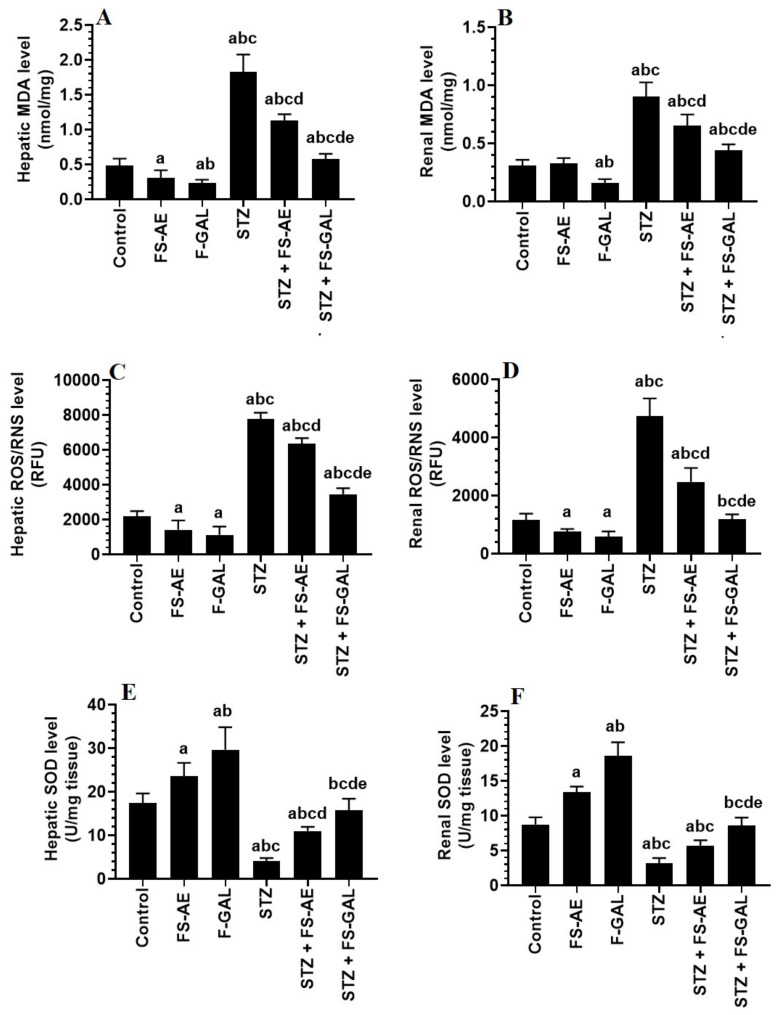
Levels of malondialdehyde (MDA) (**A**,**B**), reactive oxygen/nitrogen species (ROS/RNS) (**C**,**D**), and Superoxide Dismutase (SOD) (**E**,**F**) in the liver and kidneys of all groups of rats. All values are shown as means ± SD (*n* = 8/group). ^a^: vs. control rats, ^b^: vs. Fenugreek seed aqueous extract (FS-AE)-treated rats, ^c^: vs. Fenugreek seeds galactomannan-treated rats. ^d^: vs. streptozotocin (STZ)-diabetic rats, ^e^: vs. STZ + FS-AE-treated rats.

Levels of MDA and ROS/RNS were significantly higher, and levels of SOD were significantly lower in the livers and kidneys of STZ-treated rats as compared with the control rats (Figure 1A–E). However, there was a significant decrease in the levels of MDA and ROS with a parallel increase in the levels of SOD in the livers and kidneys of both the control and STZ-treated rats administered FS-AE or F-GAL (Figure 1A–E). However, the levels of MDA and ROS were significantly lower, and levels of SOD were significantly higher in the kidneys of F-GAL and STZ + F-GAL as compared with the FS-AE and STZ + FS-AE, respectively (Figure 1A–E). Additionally, the levels of SOD did not vary significantly, but levels of MDA and ROS remained slightly but significantly higher in the kidneys and livers STZ + F-GAL as compared to control rats (Figure 1A–E).

### 3.5. Changes in Hepatic and Renal Oxidative Stress/Antioxidant Markers

**Figure 2:**
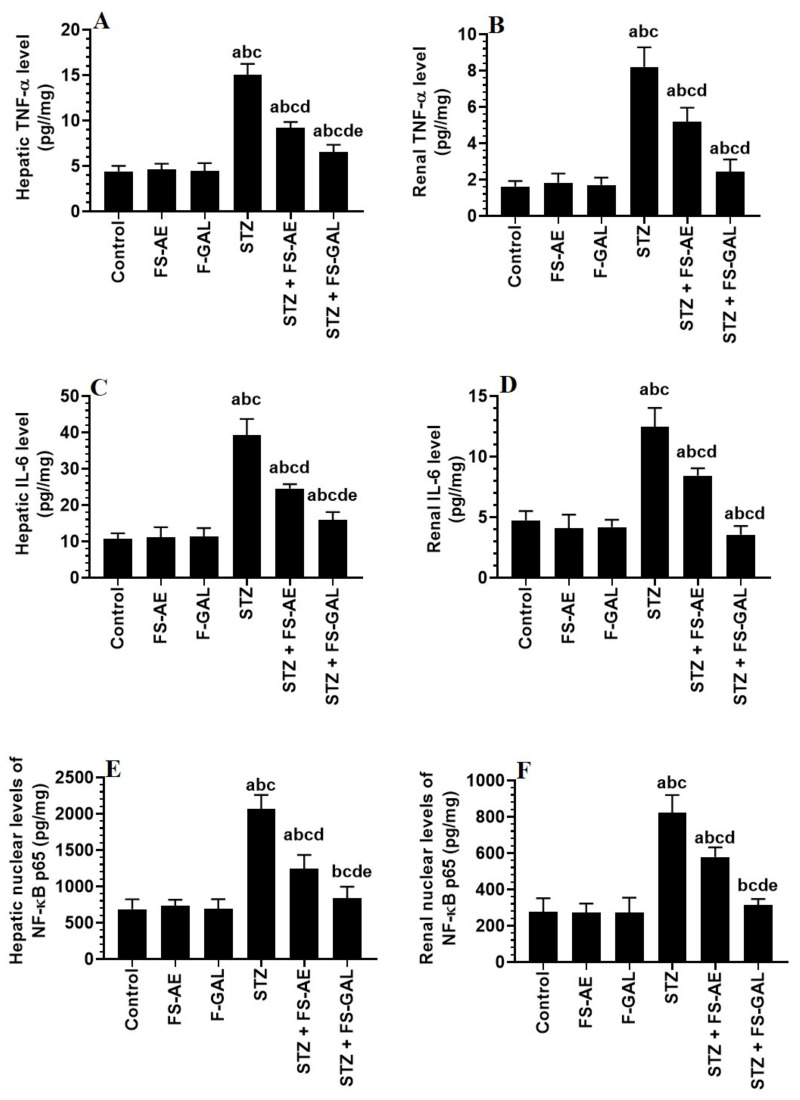
Levels of tumor necrosis-alpha (TNFα) (**A**,**B**), and interleukin-6 (IL-6) (**C**,**D**), as well as the nuclear levels of NF-κB p65 (**E**,**F**) in the liver and kidneys of all groups of rats. All values are shown as means ± SD (*n* = 8/group). ^a^: vs. control rats, ^b^: vs. Fenugreek seed aqueous extract (FS-AE)-treated rats, ^c^: vs. Fenugreek seeds galactomannan-treated rats. ^d^: vs. streptozotocin (STZ)-diabetic rats, ^e^: vs. STZ + FS-AE-treated rats.

Hepatic and renal total levels of TNF-α and IL-6 and nuclear levels of NF-κB p65 were not significantly different between the control, FS-AE, and F-GAL-treated rats (Figure 2A–F). There was a significant increase in the levels of TNF-α and IL-6, as well as nuclear levels of NF-κB in the livers and kidneys of the STZ-diabetic rats as compared to control rats which were reduced again in STZ + FS-AE and STZ + F-GAL-treated rats (Figure 2A–F). However, the levels of all these inflammatory markers were more significantly reduced in STZ + FS-AE-treated rats as compared to STZ + F-GAL-treated rats. These levels remained significantly higher than their basal levels observed in the control rats (Figure 2A–F).

### 3.6. Effect on Hepatic and Renal Keap1/Nrf2 Signaling

**Figure 3:**
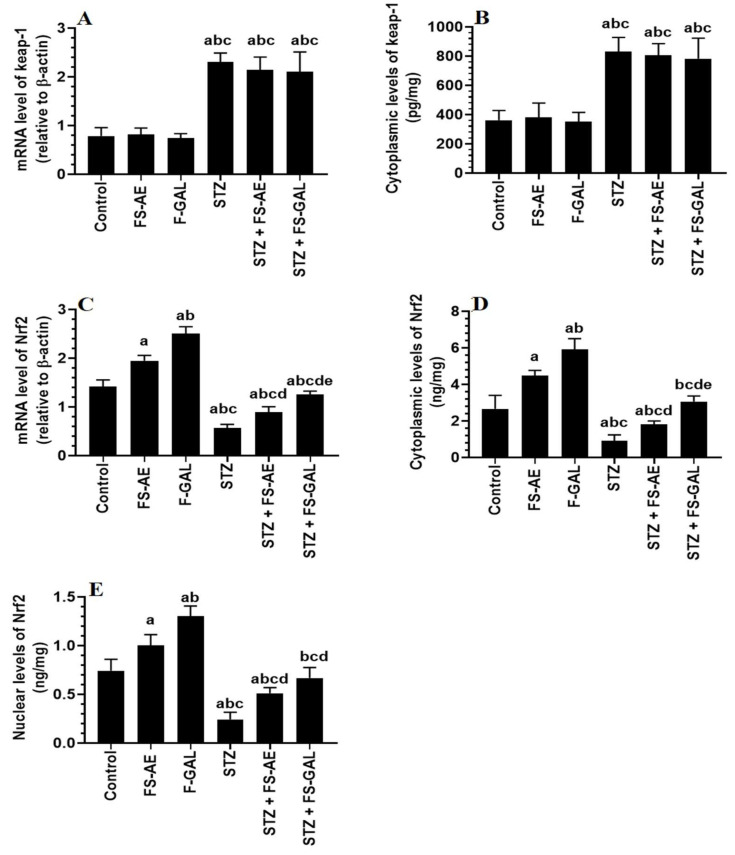
mRNA and cytoplasmic levels of keap-1 (**A**,**B**) and Nrf2 (**C**,**D**) as well as nuclear levels of Nrf2 (**E**) in the livers of all groups of rats. All values are shown as means ± SD (*n* = 8/group). ^a^: vs. control rats, ^b^: vs. Fenugreek seed aqueous extract (FS-AE)-treated rats, ^c^: vs. Fenugreek seeds galactomannan-treated rats. ^d^: vs. streptozotocin (STZ)-diabetic rats, ^e^: vs. STZ + FS-AE-treated rats.

**Figure 4:**
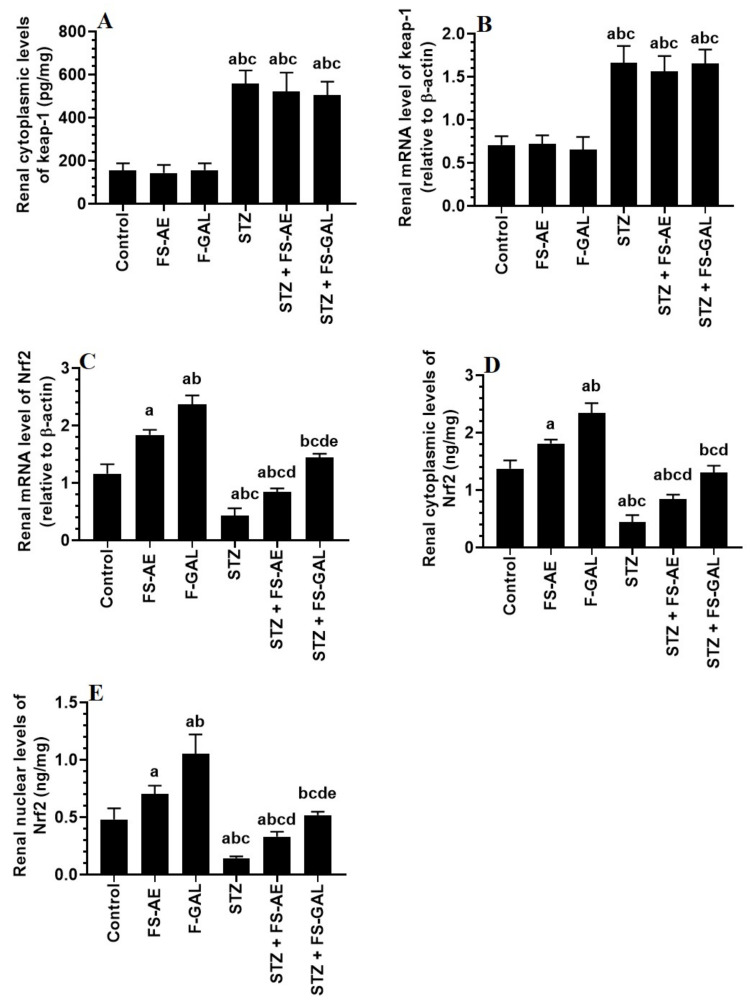
mRNA and cytoplasmic levels of keap-1 (**A**,**B**) and Nrf2 (**C**,**D**), as well as nuclear levels of Nrf2 (**E**) in the kidneys of all groups of rats. All values are shown as means ± SD (*n* = 8/group). ^a^: vs. control rats, ^b^: vs. Fenugreek seed aqueous extract (FS-AE)-treated rats, ^c^: vs. Fenugreek seeds galactomannan-treated rats. ^d^: vs. streptozotocin (STZ)-diabetic rats, ^e^: vs. STZ + FS-AE-treated rats.

mRNA and cytoplasmic levels of keap-1 were significantly increased, but mRNA, cytoplasmic, and nuclear levels of Nrf2 were significantly decreased in the livers and kidneys of the STZ-treated rats as compared to control rats (Figure 3A–E and Figure 4A–E). No significant differences in the mRNA and cytoplasmic levels of keap-1 were seen in the renal or liver tissues of the FS-AE or F-GAL as compared to control rats or in the STZ + FS-AE or STZ + F-GAL as compared to diabetic rats (Figure 3A–E and Figure 4A–E). However, mRNA, cytoplasmic, and nuclear levels of Nrf2 were significantly increased in livers and kidneys of both the FS-AE and F-GAL-treated rats as compared to control rats or in STZ + FS-AE or STZ + F-GAL as compared to diabetic rats (Figure 3A–E and Figure 4A–E). In all cases, the increments in the mRNA, cytoplasmic, and nuclear levels of Nrf2 were significantly higher in F-GAL-treated rats as compared to FS-AS-treated rats. In addition, no significant variations in the hepatic and renal mRNA, total cytoplasmic, and nuclear levels of Nrf2 were seen between the control and STZ + F-GAL-treated rats (Figure 3A–E and Figure 4A–E).

### 3.7. Histological Alterations

**Figure 5:**
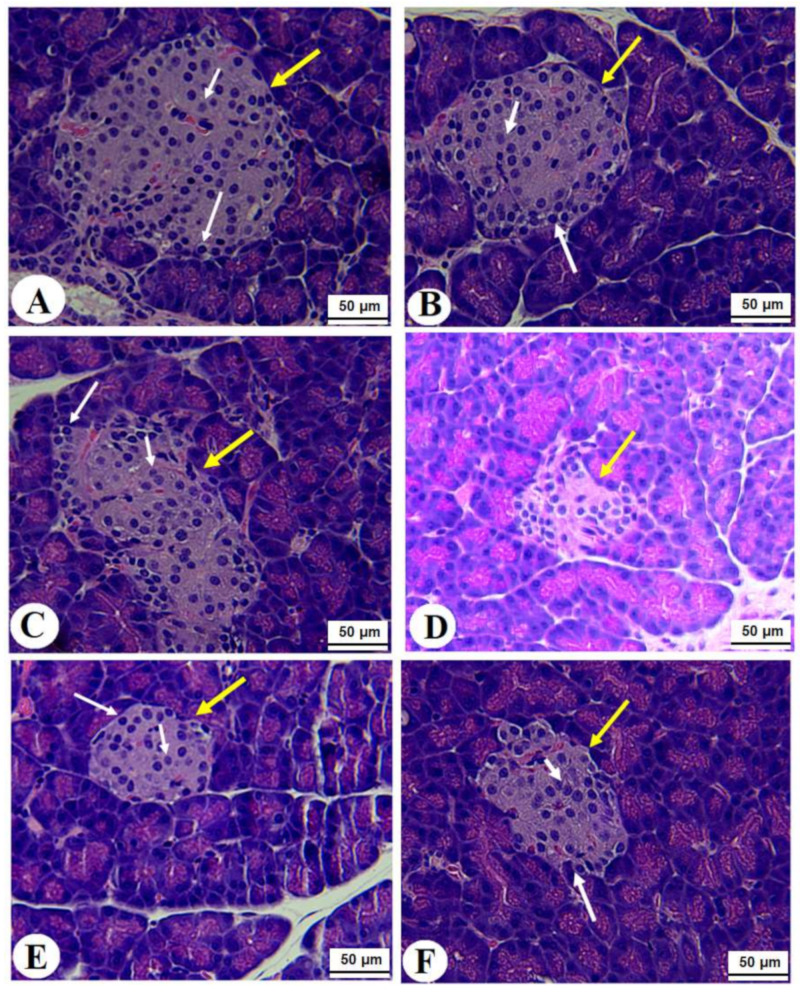
Photomicrographs of the pancreases from all groups of rats (**A**–**C**): Images taken from control, Fenugreek seed aqueous extract (FS-AE), and Fenugreek seeds galactomannan (F-GAL)-treated rats and showing normal structures of the islets of Langerhans (yellow arrow), which appear circular and of large size. The exocrine (α)-cells were located at the periphery and normally had small cells with dark nuclei (long white arrow), whereas the endocrine β-cells (short white arrow) are located centrally with larger with light nuclei. (**D**): Photomicrographs taken from a streptozotocin (STZ)-diabetic rats in which the islets of Langerhans appeared smaller (yellow arrow) with a severe reduction in the number of the α and β-cells (long and short white arrows, respectively). (**E**,**F**): represents STZ + GCS-AE and STZ + F-GAL treated rats, respectively, and shows an increase in the size of the islet of the Langerhans and the number of α (long white arrow) and β-cells β-cells (short white arrow).

**Figure 6:**
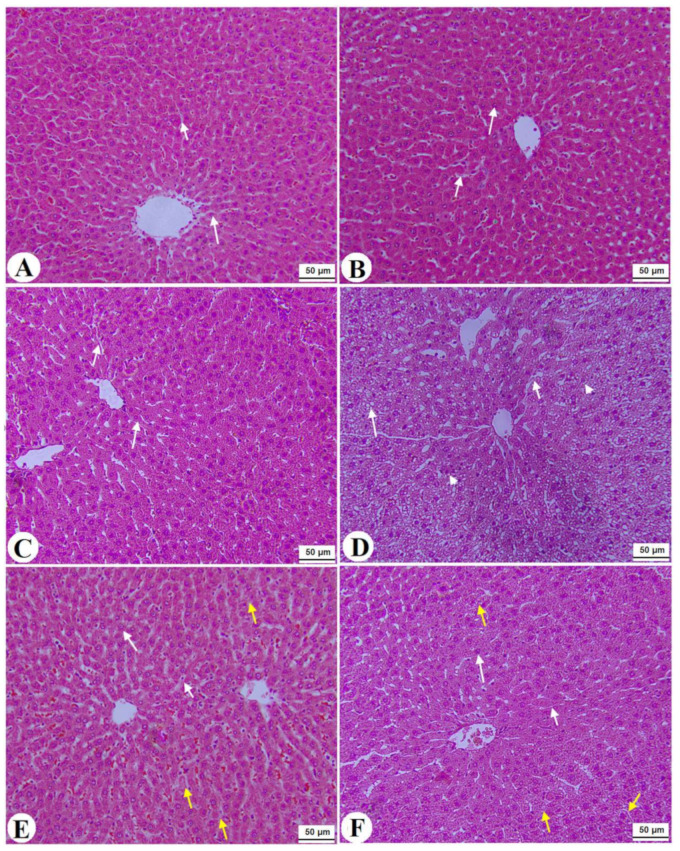
Histological images of the livers from all groups of rats (**A**–**C**): images taken from control, Fenugreek seed aqueous extract (FS-AE), and Fenugreek seeds galactomannan (F-GAL)-treated rats and showing normal liver structure with intact hepatocytes with normally rounded nuclei (long white arrow) with normally sized sinusoids (short white arrow) radiating from a central vein (CV). (**D**): was taken from a streptozotocin (STZ)-diabetic rat and showed severe fat cytoplasmic vacuoles in the hepatocyte cytoplasm of various sizes (long white arrow), dilated sinusoid (short white arrow), and an increased number of pyknotic hepatocytes (arrowhead). (**E**): Images taken from an STZ + FS-AE-treated rat and showing much improvement in the structure of the hepatocytes, where the majority of the cells and nuclei appear almost normal (long white arrow). However, some dilated sinusoids and the presence of fat vacuoles in the hepatocytes are located far from the CV (yellow arrow). (**F**): Images taken from an STZ + FS-GAL-treated rat with almost the same degree of improvement seen in T1DM + FS + AG-treated rats with almost normal hepatocytes (long white arrow) and sinusoids (short white arrow), with very few cytoplasmic fat deposits (yellow arrow).

**Figure 7:**
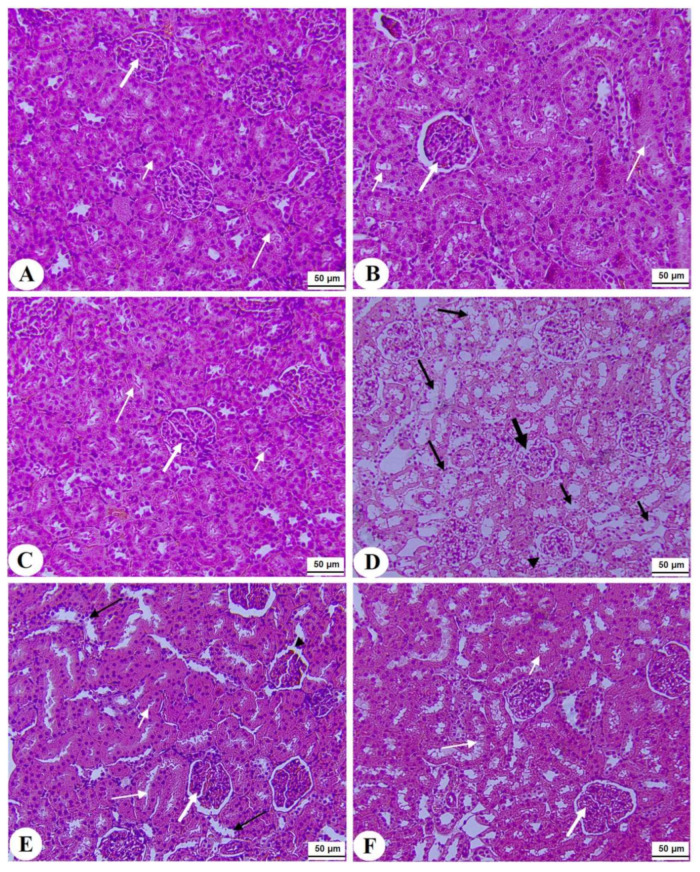
Histological section of the kidneys of all groups of rats (**A**–**C**): Histological section taken from control, Fenugreek seed aqueous extract (FS-AE), and Fenugreek seeds galactomannan (F-GAL)-treated rats and showing normal kidney morphology with intact glomerulus and glomerular membranes (long thick white arrow), proximal convolutes tubules (PCTs) (short white arrow), and distal convoluted tubules (DCTs) (long thin white arrow). (**D**): Section taken from a streptozotocin (STZ) diabetic rat and showing a reduced glomerular mass (long thick black arrow) and damaged glomerular membrane (arrowhead), PCTs (short, thin black arrow), and DCTs (long thin black arrow). (**E**,**F**): Sections taken from STZ + FS-AE and STZ + F-GAL-treated rats, respectively, and showing almost normal features, similar to those in the control rats. However, some damage in the DCTs was still seen in STZ + FS-AE-treated rats (thin black arrow). This section may be divided into subheadings. It should provide a concise and precise description of the experimental results, their interpretation, as well as the experimental conclusions that can be drawn.

Normal pancreases, liver, and kidney morphological structures were seen in the control, FS-AE, and F-GAL-treated rats (Figure 5A–C, Figure 6A–C and Figure 7A–C). Shrunk and damaged islets of Langerhans (Figure 5D) with increased loss of hepatocytes and increased fat vacuoles deposition in the hepatocytes (Figure 6D), as well as damaged glomeruli, glomerular membranes, proximal convoluted tubules (PCT), and distal convoluted tubules (DCT) (Figure 7D), were seen in STZ-diabetic rats. Improvement in the structures of all these organs was seen in the pancreases, livers, and kidneys of STZ + FS-AE and STZ + F-GAL-treated rats, with almost normal features to be seen within the STZ + F-GAL-treated rats (Figure 5A,E,F, Figure 6E,F and Figure 7E,F).

## 4. Discussion

The prevalence of DM is rapidly increasing at the global level. It is estimated that more than 400 million subjects have DM, worldwide, which is expected to increase to 600 million by the year 2040 [6]. The currently available drugs to control DM complications are the oral hypoglycemic agent and insulin, which are still expensive and are associated with many side effects [50]. Therefore, the surge in searching for cheaper and safer therapies from plant resources is under focus. Previous experiments and clinical studies have identified Fenugreek seeds (FS) as a possible treatment for DM and some of its complications due to its documented hypoglycemic, hypolipidemic, and antioxidant potentials. In the current investigation, we are also confirming these effects and further demonstrating the ability of FS-AE and its active ingredient to alleviate STZ-induced hypoinsulinemia, pancreatic-β cell damage, diabetic nephropathy, liver damage, and hepatic steatosis. Our data suggest that scavenging ROS, upregulation of antioxidants, reducing inflammatory cytokines production, inhibiting NF-κB p65, and upregulation and activation of Nrf2 are the major interconnected mechanisms underlying these protective effects of both FS-AE and F-GAL. Interestingly, we have also identified F-GAL to exert more powerful protection as compared to FS-AE.

Insulin is the most metabolic hormone responsible for glucose and lipid hemostasis through stimulating cellular glucose, amino acids, and fatty acids (FAs) uptake, as well as protein, glycogen, and lipid synthesis [51]. STZ-induced T1DM in rats and mice is considered the most common animal model to study the pathogenesis of the disease and the discoveries of new therapies which induce oxidative pancreatic β-cells damage (60–80%) through generating localized ROS RNA, and carbonium radicals [52]. In addition, STZ animal models exhibit similar clinical manifestations that are very close to those seen in human subjects, including adipose tissue lipolysis, muscle wasting, weight loss, polyphagia, fasting hyperglycemia, hypoinsulinemia, polyuria, and polydipsia [52]. Yet, the HOMA-β index remains the most acceptable theoretical index that positively quantifies β-cell function and mass [53]. All these clinical signs with clear histological damage of β-cells, as well as a reduction in the values of the HOMA-β index, were also observed in our STZ-induced T1DM animal model of this study, thus validating it.

However, the obvious reduction in fasting hyperglycemia and the improvements in circulatory insulin levels and levels of HOMA-β in STZ + FS-AE and STZ + F-GAL-treated rats were our clearest evidence for the hypoglycemic effect of the FS-AE and F-GAL which are indicated to be a result of promoting the generation of pancreatic β-cells. This has also been confirmed by the improvement in the structure of the β-cells in the pancreases of these groups of rats. However, the effects were more profound within the rats that were administered F-GAL as compared to FS-AE. As discussed later and given that both FS-AE and F-GAL attenuated STZ-induced liver and renal damage by attenuating oxidative stress and suppressing inflammation, at this stage, we may explain that the induction of β-cell mass by both extracts is, at least, mediated by the antioxidant and anti-inflammatory effects of both extracts. In addition, and without any alteration in circulatory levels of insulin, there was a significant reduction in fasting glucose levels with a significant increment in the levels of HOMA-β in both FS-AE and F-GAL-treated control rats. These data may indicate the existence of other mechanisms of action, such as reducing carbohydrate digestion and glucose absorption, suppressing hepatic glucose output, and stimulating peripheral glucose uptake. Unfortunately, this was not investigated in this study and requires further research.

These data support previous studies that have also reported the hypoglycemic effect of various extracts and GAL fraction of FS. In support, the hypoglycemic effects of ethanol, methanol, and water extracts of FS were reported and explained by acting through different mechanisms, including suppressing intestinal glucose digestion and absorption, correcting insulin-sensitive carbohydrate metabolic enzymes involved in gluconeogenesis and glycogen synthesis (e.g., hexokinase, glucose-6-phosphatase, and glucose-6-phosphate dehydrogenase), increasing peripheral glucose disposal, improving insulin action, and decreasing β-cell damage through suppressing oxidative stress and inflammation and stimulating antioxidant enzymes [28,29]. However, other authors have shown that the hypoglycemic effect of FS is mainly due to generation due to the presence of 4-hydroxy isoleucine, a novel amino acid known to stimulate β-cell generation, insulin secretion, and peripheral glucose uptake and insulin signaling [54,55]. On the contrary, other authors have shown that the active ingredient of FS, diosgenin, is the most important agent that reduces fasting hyperglycemia in diabetic animals promoting the β-cells generation, inhibiting hepatic gluconeogenesis, and stimulating liver glycolysis (hepatic glucokinase) [55,56]. 

However, the potent hypoglycemic and insulin-releasing potentials of F-GAL are following the findings of Anwar et al. [57] and Kamble et al. [36], who have similar effects but in alloxan diabetic mice and explained it by attenuating the degeneration of β-cells through upregulating ameliorating pancreatic lipid peroxidation and antioxidants enzymes CAT, SOD, and GPx. Furthermore, Srichamroen et al. [58] have shown that the hypoglycemic effect of F-GAL in diabetic rats is also mediated by inhibiting intestinal glucose digestion and absorption via suppressing the digestive enzymes, amylase, and sucrose. In contrast, other authors have shown that the hypoglycemic effect triggered by F-GAL in animal models of T1DM and T2DM occurs through reducing glucose absorption and improving peripheral insulin action, respectively, but independent of modulating pancreatic insulin secretion [34,35,36].

On the other side, hepatic and renal damage are the two major early complications seen in T1DM patients and the STZ-treated diabetic animal model [8]. Overproduction of ROS has been documented as the major mechanism responsible for hepatic and renal damage by triggering DNA fragmentation, lipid peroxidation, inflammation, fibrosis, and apoptosis [1,59]. Within this context, hyperglycemia promotes massive cellular production of ROS in these tissues by stimulating glucose auto-oxidation, scavenging GSH, glycation, and suppression of antioxidant enzymes, impairing the mitochondrial oxidative phosphorylation, ketoacidosis, and activating several ROS-generating pathways, including AGEs, hexosamine phosphate, and PKC)/NADPH oxidase pathway [29]. In turn, ROS promotes liver and kidney damage by depleting GSH and antioxidants, activating NF-κB, upregulating inflammatory cytokines, activating the transforming growth factor-β1 (TGF-β1) signaling, and inducing intrinsic (mitochondria-mediated) cell apoptosis [10,11]. However, Nrf2 and NF-κB are two transcription factors related to cell antioxidant potential (22). Currently, accumulating data have shown that sustained hyperglycemia and oxidative stress exaggerate the hepatic and renal damage in diabetic animals by downregulating Nrf2 transcription and stimulating keap-1-induced degradation of Nrf2, a mechanism that leads to stimulating NF-κB and suppressing GSH and phase-II antioxidant enzyme [17].

In this study, the significant increase in the levels of liver function enzymes, ALT and AST; the significant reduction in serum albumin; and the increase in serum urea and creatinine, as well as the obvious damage in the structure of the hepatocytes and renal tubules, indicate that STZ-induced diabetes in rats is associated with hepatic and renal damage, which supports many other previous studies which utilized the same STZ-rat model [8,60,61]. In addition, and in support of the above-mentioned studies, oxidative stress and inflammation were evidenced in the kidneys and livers of the diabetic rats of this study as marked by increased generation of ROS and MDA, increased nuclear levels of NF-κB, upregulation of TNF-α and IL-6, upregulation of keap-1 mRNA and cytoplasmic levels, and reduced transcription, total cytoplasmic, and nuclear levels of Nrf2. Opposing this, the administration of both FS-AE and F-GAL reversed all these mechanisms and improved the structure of the liver and kidneys of diabetic rats. They also stimulated mRNA, total cytoplasmic, and total nuclear levels of Nrf2 in the livers and kidneys of control rats, which coincided with higher levels SOD. Of note, administration of FS-AE and F-GAL did not alter the expression or levels of keap-1. Again, the effects afforded by F-GAL on all of these oxidant, antioxidant, and inflammatory markers, as well as the histological features of the livers and the kidneys, were more powerful than those exerted by FS-AE. Based on these data, we have become more confident that the hepatorenal anti-inflammatory protection afforded by FS-AE and F-GAL is mediated by its potent antioxidant potential and its ability to upregulate antioxidants through upregulating/activating the Nrf2/antioxidant axis.

Although some studies have examined the nephroprotective effect of FS in diabetic rodents, the hepatoprotective effects of these extracts were poorly investigated and were targeted in this study. Indeed, a previous study has shown that FS-AE could alleviate diabetic nephropathy in rats by increasing glycosaminoglycan heparin sulfate (HS) and suppressing collagen deposition [30]. A more recent study has also revealed that supplementation with FS-AE (mainly GAL) protected against STZ-induced diabetic nephropathy and fibrosis in rats by suppressing lipid peroxidation and increasing GPx levels [31]. However, previous studies have shown that the antioxidant and anti-inflammatory effects of FS extracts could be due to the high content of diosgenin, a potent antioxidant and anti-inflammatory agent that can stimulate antioxidant enzymes and suppress inflammatory cytokines production reviewed in [55]. Our data also extend this and suggest that the F-GAL is a significant contributing antioxidant and anti-inflammatory molecule that could act similarly. In support, F-GAL prevented liver and renal damage in STZ-diabetic rats with no clear mechanism. However, our data are still unique. They further show that these renal and hepatic protections afforded by FS-AE and F-GAL are also concomitant with upregulation and activation of Nrf2, which could underlie their stimulatory effect on antioxidant enzymes that have been described in many previous studies. Supporting our data, administration of the glycosides-based standardized FS-AE also suppressed pulmonary inflammation, fibrosis, and apoptosis by activating Nrf2-mediated suppression of NF-κB p65 and Bax [32].

Although these effects can be observed as secondary effects of the hypoglycemic and anti-diabetic effects of FS-AE and F-GAL, the available data obtained from the toxicity studies indicate that both FS-AE and F-GAL have antioxidants and anti-inflammatory effects that are independent of their hypoglycemic potential. Indeed, administration of FS-AE or ethanol extract attenuated liver damage in aluminum chloride (ALCl3), thioacetamide, and ethanol-treated rats and human Chang cell culture by reducing lipid peroxides (MDA or TBARS), ameliorating the distribution of iron metabolism, increasing levels of GSH, GPx, SOD, and glutathione-reductase (GRx) [23,62]. Aloes, FS powder, or ethanol extract repressed dieldrin and carbon tetrachloride (CCL4)-induced renal and hepatic toxicity in rats by reducing TBARS and a parallel increase in the levels of GSH SOD and GPx [63]. The FS-AE also reduced ethylene glycol-induced kidney stones by inhibiting MDA levels [64].

Nonetheless, hyperglycemia associated with T1DM and T2DM is also associated with hyperlipidemia and hepatic steatosis, an effect that is attributed to increased mobilization of FFAs from the adipose tissue due to lack of insulin [4,5]. Furthermore, hyperglycemia, ROS, and inflammatory cytokines mediate hepatic steatosis by uncontrolled stimulation of the lipogenic transcription factors SREBP1/2 and their target CHOL, as well as TGs synthesis genes, and concomitant suppression of PPARα, which is responsible for the mitochondrial FAs (β)-oxidation [65]. Another interesting observation in this study is the ability of both FS-AE and F-GAL to alleviate the increase in serum levels of FFAs, TGs, CHOL, and LDL-c, reduce hepatic serum levels of TGS, CHOL, and FFAs, and reduce hepatic steatosis in the livers of diabetic rats. However, our data confirm that F-GAL treatment is more powerful in reducing hyperlipidemia and hepatic steatosis in rats rather than FS-AE. These results are not novel and have been previously documented for FS different extracts, as well as for F-GAL in previous research [22,58]. Within this view, studies have shown that FS contains some active hypolipidemic ingredients, including diosgenin and 4-hydroxy isoleucine, which mediates its lipid-lowering effect [55]. Indeed, diosgenin reduced serum TGs, CHOL, and LDL-c, stimulated circulatory HDL-c levels, and suppressed hepatic lipid accumulation and deposition in HepG2 cells and HFD-fed animals by downregulating SREBP1c and stimulating PPARα, two transcription factors that promote TGs synthesis and FAs oxidation, respectively [66].

On the other hand, the inhibitory effect of F-GAL in HFD-fed diabetic animals was attributed to decreasing the intestinal absorption of LDL-c and CHOL. However, an interesting observation in this study is that the inhibitory effect of FS and F_GAL on serum and hepatic lipids were only observed in the livers of diabetic rats, thus suggesting it is a secondary effect due to its hypoglycemic, antioxidant, and anti-inflammatory effects. This remains observational and further studies are needed to confirm this assumption.

However, despite these data, our study still has an important limitation. Although we have shown a stimulatory effect on FS-AE and F-GAL on the transcription and activation of Nrf2, whether activation of Nrf2, the upstream mediating, is the hepatorenal protective effect of these extracts remains observational. We need further studies utilizing cells or animals deficient with Nrf2 to confirm these events. In addition, we need further examination to confirm further mechanisms responsible for the hypoglycemic and hypolipidemic effects of FA-AE and F-GAL, such as their effect on glucose and lipid digestion and absorption and their effect on hepatic gluconeogenic and lipogenic genes. Furthermore, the biochemical analysis used to measure MDA could be inaccurate. Further examinations such as monitoring the levels of 4HNE and other antioxidants such as GSH, GPx, thioredoxin, etc., should be considered in further studies.

## 5. Conclusions

Our data uniquely describe and compare the hepatorenal protective effect of FS-AE and F-GAL in STZ-diabetic animals. The summary of these data indicates the ability of both extracts to alleviate STZ-induced liver and renal damage through their concomitant hypoglycemic and antioxidant effects that involve upregulation of Nrf2.

## Figures and Tables

**Table 1:** Primer characteristics of the real-time PCR.

Gene	Primers (5′→3′)	GenBank Accession	Product Length
Nrf2	F: -AAAATCATTAACCTCCCTGTTGAT R: R: ′-CGGCGACTTTATTCTTACCTCTC	NM_031789	118
Keap-1	5′-TATGAGCCAGAGCGGGACGA-3′ 5′-TCATCCGCCACTCATTCCTCT-3′	AF304364.1	172
B-actin	F: GACCTCTATGCCAACACAGT R: CACCAATCCACACAGAGTAC	NM_031144	154

**Table 2:** Final body and organ weights, food intake, and fasting levels of glucose and insulin in all groups of rats.

	STD	FS-AE	F-GAL	STZ	STZ + FS-AE	STZ + F-GAL
Final body weight (g)	418 ± 22	422 ± 26	409 ± 32	313 ± 19 ^abc^	379 ± 18 ^abcd^	411 ± 20 ^de^
Weekly food intake (g/rat) (Last 2 weeks)	192 ± 16	185 ± 22	198 ± 23	274 ± 28 ^abc^	217± 15 ^abcd^	186 ± 25 ^de^
Liver weight (g)	14.7 ± 1.1	13.1 ± 1.6	14.7 ± 1.2	18.7 ± 2.0 ^abc^	16.4 ± 0.8 ^abcd^	14.2 ± 1.1 ^de^
Kidney weight (g)	1.46 ± 0.23	1.49 ± 0.32	1.36 ± 0.29	1.03 ± 0.18 ^abc^	1.24 ± 0.23 ^abcd^	1.44 ± 0.22 ^de^
Plasma fasting glucose (mg/dL)	105.6 ± 7.7	91.3 ± 6.3 ^a^	87.1 ± 5.7 ^a^	302.1 ± 34 ^abc^	174.6 ± 21 ^abcd^	124.5 ± 12.1 ^abcde^
Plasma fasting insulin (µIU/mL)	5.5 ± 0.53	5.1 ± 0.62	5.3 ± 0.59	1.8 ± 0.33 ^abc^	2.87 ± 0.46 ^abcd^	3.41 ± 0.63 ^abcde^
HOMA-β index	47.2 ± 6.3	66.5 ± 6.6 ^a^	76.5 ± 9.2 ^ab^	2.8 ± 0.53 ^abc^	9.8 ± 1.1 ^abcd^	22.1 ± 3.5 ^abcde^

All values are shown as means ± SD (*n* = 8/group). ^a^: vs. control rats, ^b^: vs. Fenugreek seed aqueous extract (FS-AE)-treated rats, ^c^: vs. Fenugreek seeds galactomannan-treated rats. ^d^: vs. streptozotocin (STZ)-diabetic rats, ^e^: vs. STZ + FS-AE-treated rats.

**Table 3:** Serum and hepatic lipid profile in all groups of rats.

	STD	FS-AE	F-GAL	STZ	STZ + FS-AE	STZ + F-GAL
Serum						
TGs (mg/dL)	54.5 ± 5.4	58.9 ± 7.2	55.3 ± 8.8	121 ± 11.4 ^abc^	85.4 ± 7.5 ^abcd^	61.2 ± 5.9 ^acde^
CHOL (mg/dL)	77.5 ± 9.4	83.3 ± 7.5	79.5 ± 8.1	143 ± 12.3 ^abc^	104 ± 3.2 ^abcd^	81.4 ± 6.9 ^de^
LDL-c (mg/dL)	33.2 ± 4.3	29.6 ± 4.1	31.5 ± 4.8	71.5 ± 7.7 ^abc^	53.1 ± 6.8 ^abcd^	35.7 ± 5.4 ^bcde^
HDL-c (mg/dL)	24.5 ± 4.3	35.2 ± 5.1	23.7 ± 4.8	11.1 ± 2.3 ^abc^	17.8 ± 2.7 ^abcd^	25.4 ± 3.1 ^de^
FFAs (µmol/L)	224 ± 14.5	216 ± 19.4	227 ± 17.6	646 ± 35.7 ^abc^	342 ± 24.7 ^abcd^	271 ± 19.7 ^abcde^
Liver						
Triglycerides (µg/g)	4642 ± 220	4593 ± 198	4721 ± 318	9135 ± 615 ^abc^	6619 ± 472 ^abd^	5492 ± 301 ^abcde^
CHOL (µg/g)	1594 ± 152	1499 ± 178	1518 ± 159	4021 ± 328 ^abc^	2821± 219 ^abcd^	1612 ± 171 ^bcde^
FFAs (µmol/g)	63.7 ± 7.5	59.6 ± 5.9	62.5 ± 6.9	125.1 ± 10.5 ^abc^	89.3 ± 6.8 ^acbd^	71.8 ± 6.6 ^abcde^

All values are shown as means ± SD (*n* = 8/group). ^a^: vs. control rats, ^b^: vs. Fenugreek seed aqueous extract (FS-AE)-treated rats, ^c^: vs. Fenugreek seeds galactomannan-treated rats. ^d^: vs. streptozotocin (STZ)-diabetic rats, ^e^: vs. STZ + FS-AE-treated rats.

**Table 4:** Levels of kidney and liver function markers in all groups of rats.

	STD	FS-AE	F-GAL	STZ	STZ + FS-AE	STZ + F-GAL
ALT (U/L)	34.3 ± 3.5	35.6 ± 4.1	32.5 ± 3.9	79.7 ± 7.4 ^abc^	53.4 ± 6.3 ^abcd^	38.9 ± 3.5 ^abcde^
AST (U/L)	48.7 ± 5.3	41.5 ± 6.4	47.6 ± 5.9	132.1 ± 9.5 ^abc^	86.5 ± 8.7 ^abcd^	55.6 ± 5.1 ^abcde^
Albumin (g/dL)	5.9 ± 0.93	6.1 ± 1.2	5.5 ± 0.89	2.3 ± 0.43 ^abc^	4.3 ± 0.69 ^abcd^	5.8 ± 0.83 ^de^
Urea (mg/dL)	4.3 ± 0.47	4.1 ± 0.61	3.9 ± 0.42	9.3 ± 1.7 ^abc^	6.5 ± 1.4 ^abcd^	4.2 ± 0.79 ^de^
Creatinine (mg/dL)	0.73 ± 0.04	0.69 ± 0.05 ^a^	0.64 ± 0.06	2.56 ± 0.71 ^abc^	1.25 ± 0.16 ^abcd^	0.95 ± 0.11 ^abcde^

All values are shown as means ± SD (*n* = 8/group). ^a^: vs. control rats, ^b^: vs. Fenugreek seed aqueous extract (FS-AE)-treated rats, ^c^: vs. Fenugreek seeds galactomannan-treated rats. ^d^: vs. streptozotocin (STZ)-diabetic rats, ^e^: vs. STZ + FS-AE-treated rats.

## Data Availability

The datasets used and analyzed during the current study are available from the corresponding author on reasonable request.
